# Eating Frequency Is Not Associated with Obesity in Chinese Adults

**DOI:** 10.3390/ijerph15112561

**Published:** 2018-11-15

**Authors:** Xinge Zhang, Yueqiao Wang, Jason S. Brinkley, Theresa M. Oniffrey, Rui Zhang, Guoxun Chen, Rui Li, Justin B. Moore

**Affiliations:** 1 School of Health Sciences, Wuhan University, Wuhan 430071, China; ylcz2920@126.com (X.Z.); elle529@126.com (Y.W.); 2 Abt Associates, Durham, NC 27703, USA; Jason_brinkley@abtassoc.com; 3 Cerus Consulting, LLC, Winston-Salem, NC 27101, USA; toniffrey@gmail.com; 4 College of Life Sciences, South-Central University for Nationalities, Wuhan 430074, China; zhangrui@mail.scuec.edu.cn; 5 Department of Nutrition, the University of Tennessee at Knoxville, Knoxville, TN 37996, USA; gchen6@utk.edu; 6 Department of Family & Community Medicine, Wake Forest School of Medicine, Wake Forest Baptist Medical Center, Medical Center Boulevard, Winston-Salem, NC 27157, USA; 7 Department of Epidemiology & Prevention, Wake Forest School of Medicine, Bowman Gray Center, 475 Vine St, Winston-Salem, NC 27101, USA; 8 Department of Implementation Science, Wake Forest School of Medicine, Bowman Gray Center, 475 Vine St, Winston-Salem, NC 27101, USA

**Keywords:** eating frequency, adiposity, sociodemographic factors, lifestyle factors, nutrition knowledge

## Abstract

The prevalence of overweight and obesity has been increasing globally. Recent studies suggest that eating frequency (EF) might be a factor influencing the development of overweight and obesity. This study aims to explore the association between eating frequency and obesity in Chinese adults. A cross-sectional study was conducted in Wuhan, China, from March to June 2016. A self-administered questionnaire and 24-h dietary recall were used to collect data on sociodemographic variables, lifestyle factors, nutrition knowledge, and eating frequency. Participants were divided into four groups according to eating frequency and meal timing: traditional time pattern (TTP), traditional time plus late snack pattern (TTLSP), irregular time pattern (ITP), and all-day pattern (ADP). We performed the chi-squared test and multiple logistic regression to assess associations among variables using JMP statistical software version 14.0.0 (SAS Institute Inc., Cary, NC, USA). Respondents were Chinese adults (*N* = 2290; range 29–74 years; 1162 men). Lower education level, higher food budget, and lower nutrition knowledge were associated with higher likelihood of irregular EF patterns (TTLSP, ITP, or ADP). Men, non-smokers, and participants with less physical activity, lower education level, or lower nutrition knowledge were more likely to be obese. Body mass index (BMI) categorization was significantly different among EF pattern groups (χ^2^ = 25.40, *p* = 0.003); however, this association was no longer significant in the regression model after adjustment for age, sex, education, smoking, food budget, nutrition knowledge, and physical activity. Thus, EF is not associated with obesity in Chinese adults.

## 1. Introduction

The prevalence of being overweight and obesity has been increasing globally. As reported by the World Health Organization, more than 1.9 billion adults were overweight in 2016, and of these, over 650 million were obese [1]. In China, in 2013, 28.3% of men and 27.4% of women aged ≥20 years old were overweight or obese [2]. Overweight and obesity are associated with increased risk of many serious diseases and health conditions, including cardiovascular diseases [3], osteoarthritis [4], and certain cancers [5]. The fundamental cause of obesity and overweight is an energy imbalance between calories consumed and calories expended [2]. As a determining factor of energy intake, eating frequency (EF) has been suggested to be a factor potentially influencing overweight and obesity [6].

EF is defined as the number of eating occasions per day; commonly in research contexts, an eating occasion is defined as any instance in which participants report consumption of a meal or a snack, with a minimum gap of 15 min set between eating occasions (or they are counted as the same occasion) [7,8]. In 1964, Fábry et al. reported that lower EF might increase body weight [6]. Subsequently, many epidemiologic studies have been conducted to investigate the association between EF and body weight; however, the results have been highly inconsistent. Previous studies have observed inverse [9,10,11,12,13,14], null [15,16,17], and positive [18,19] associations of EF with overweight and obesity. Murakami et al. suggested that the inconsistencies were caused by different methods of assessing EF [18]. Currently, four different definitions of EF are commonly employed in surveys assessing meal and snack frequency based upon (1) contribution to total energy intake [20], (2) time of day [21], (3) self-reported eating occasions [22], and (4) neutral definition [23]. Lack of adjustment for potentially influential factors also contributed to the inconsistencies; potential confounding roles of physical activity, socioeconomic status, smoking, and nutrition knowledge have been reported [18,24,25,26,27,28,29]. Although several studies along these lines have been conducted in Western populations, studies conducted in eastern Asian populations are still lacking.

Therefore, the objective of the present investigation is to explore the associations between EF and overweight/obesity in Chinese adults, focusing on the influences of adjustment for nutrition knowledge, sociodemographic, and lifestyle factors.

## 2. Materials and Methods 

### 2.1. Study Design

This cross-sectional study was conducted in Wuhan, China, from March to June 2016. A self-administered questionnaire was reviewed by a panel of experts and then piloted online for comprehensibility in April 2016. In the pretest, a convenience sample of 113 individuals completed the survey and the questions were evaluated by an expert panel for content, length, and readability. Forty-three questions were included in the final version. In the first week of May 2016, a revised questionnaire was distributed to adults whose children were studying at the No. 1 Jiangxia High School or the No. 4 Jiangxia Middle School in Wuhan, China; participants responded to the questionnaire voluntarily, without any incentives. Written informed consent was obtained from the participants before the completion of the survey. The study was conducted in accordance with the Declaration of Helsinki, and all procedures were approved by the Wuhan University Ethics Board (ethical approval code: 2016031270) and the school district administrators.

A total of 2824 adults consented to participate in the survey, of whom 2290 adults (aged 29–74; 1162 men and 1128 women) were included in the analytical sample. We excluded individuals according to the following criteria: BMI scores ± 2 z-scores above or below the normal range (i.e., BMI < 15.43 kg/m^2^ or BMI > 32.00 kg/m^2^); non-viable answers on the nutrition knowledge questions (e.g., answering “A” on all items).

### 2.2. Assessment of Sociodemographic Characteristics

Demographic information was collected by self-report. Age was divided into 29–33, 34–38, 39–43, 44–48, 49–53, 54–58, 59–63, 64–68, and 69–73-year-old groups, while educational attainment was divided into primary school, middle school, high school, some college, bachelor’s degree, and graduate degree groups. Information on current smoking status (yes or no) and weekly food budget was also collected. In 2013, the Engle’s Coefficient (food expenditures/total living expenditures) of China was 35% [30], and the annual average food cost of city residents was 26,955 RMB; therefore, the average monthly food budget was 786 RMB. We categorized food budgets into less than 500 RMB, 500–1000 RMB, 1000–1500 RMB, and more than 1500 RMB monthly.

### 2.3. Assessment and Definition of EF

EF was defined based upon the time of day participants reported eating meals [21]. We used the time intervals and local eating time traditions to define whether EF pattern was regular or not. A 24-h diet recall method was applied, in which participants were asked to report meals and snacks eaten between the hours of 600–1000, 1000–1100, 1100–1400, 1400–1600, 1600–2000, and 2000–600. Eating only between 600–1000, 1100–1400, and 1600–2000 was defined as the traditional time pattern (TTP). The traditional time plus late snack pattern (TTLSP) was assigned to people who ate meals at the TTP but also ate a snack between 2000 and 0600. The all-day pattern (ADP) was people who ate in each of the six-time blocks. The irregular time pattern (ITP) covered all people who did not fall into the other categories.

### 2.4. Evaluation of Nutrition Knowledge

Since there is no widely used or validated scale to evaluate nutrition knowledge in China, we designed a questionnaire based on previous Chinese research examining nutritional knowledge, attitudes, and practice [31]. Thirteen nutrition questions, pilot-tested for readability and content validity, were included in our questionnaire. A score of 1 was assigned when an answer was correct; correct answers were summed, and nutrition knowledge was categorized based on the total score (0–6 for low, 7–10 for moderate, and 11–13 for high levels of nutrition knowledge). Participant data were used only if they reported on a separate question that their responses represented their typical eating pattern.

### 2.5. Assessment of Overweight/Obesity and Physical Activity

Body mass index (BMI) was calculated by self-reported height (kg) and weight (m^2^), with overweight and obesity respectively defined as BMI ≥ 24.0 kg/m^2^ and ≥ 28.0 kg/m^2^, according to Chinese national standards [32]. Physical activity intensity was categorized according to how many days per week the respondent reported being engaged in high-intensity physical activities; examples given were running and jogging, hiking, riding a bicycle at a high speed, and competitive sports. Responses were dichotomized: participants who reported vigorous physical activity at least 4 days/week were considered to have an active lifestyle.

### 2.6. Statistical Analysis

Statistical analyses were performed using JMP statistical software, version 14.0.0 (SAS Institute Inc., Cary, NC, USA). Descriptive statistics were used to describe the distribution of the variables. The chi-squared test was conducted to compare Overweight/Obesity (BMI ≥ 24.0 kg/m^2^), nutrition knowledge, and sociodemographic, and lifestyle factors among EF patterns. Nominal logistic regression models were carried out to explore the potential influences on EF patterns. The association between EF pattern and BMI group was analyzed using models adjusted for sex, age, smoking, education, physical activity, food budget, and nutrition knowledge. Two-tailed statistical significance was set at *p* < 0.05.

## 3. Results

### 3.1. Demographic Information

Characteristics of participants are summarized in Table 1. Most participants were between 29–59 years old. Nearly 50% of men reported smoking. Most respondents were distributed between two food budget categories (≤1000 RMB/month or >1000 RMB/month). Over three-quarters of participants reported that they did not engage in regular vigorous physical activity (≥4 days per week). Only 27% of the participants had a high level of nutrition knowledge, and about 28% of participants were either overweight or obese. Respectively, 48%, 30%, 10%, and 12% of participants ate with TTP, ITP, TTLSP, and ADP.

### 3.2. Differences in BMI Distribution, Nutrition Knowledge, and Food Budget among EF Patterns

Table 2 shows a crosstabulation describing BMI distribution across different EF patterns. The percentage of respondents who were obese by EF pattern was 12.8% for ADP, 11.4% for TTLSP, 9.9% for ITP, and 9.5% for TTP. The difference in BMI distribution among EF patterns was statistically significant (χ^2^ = 25.40, *p* = 0.003). Table 3 shows that low nutrition knowledge rates increased, and high rates decreased in the above order. The difference in nutrition knowledge among EF patterns was significant (χ^2^ = 152.81, *p* < 0.001). There was a significant difference in food budget across EF patterns (χ^2^ = 59.83, *p* < 0.001). Notably, a greater percentage of participants spent more than 1500 RMB on foods from TTP to ADP (Table 4). 

### 3.3. Associations among EF Pattern, Nutrition Knowledge, Sociodemographic, and Lifestyle Factors and BMI Group

**Figure 1:**
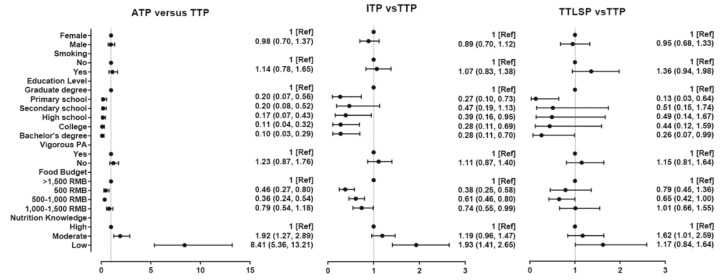
Associations between nutrition knowledge, sociodemographic, and lifestyle factors and eating frequency (EF).

The association between EF pattern and demographic factors is shown in Figure 1 and Table S1. Odds ratios (ORs) with 95% confidence intervals (CIs) for ADP from “Primary school” to “Bachelor’s degree” were respectively 0.20 (0.07, 0.56), 0.20 (0.08, 0.52), 0.17 (0.07, 0.43), 0.11 (0.04, 0.32), and 0.10 (0.03, 0.29). The same tendency also presented when ITP and TTLSP were compared with TTP: ORs with 95% CIs for ITP were respectively 0.27 (0.10, 0.73), 0.47 (0.19, 1.13), 0.39 (0.16, 0.95), 0.28 (0.11, 0.69), 0.28 (0.11, 0.70), and for TTLSP, 0.13 (0.03, 0.64), 0.51 (0.15, 1.74), 0.49 (0.14, 1.67), 0.44 (0.12, 1.59), 0.26 (0.07, 0.99). People with lower food budgets were more likely to have TTP rather than ITP: ORs with 95% CIs for ADP from “Less than 500 RMB” to “1000–1500 RMB” were respectively 0.46 (0.27, 0.80), 0.36 (0.24, 0.54), and 0.79 (0.54, 1.18), while for ITP they were respectively 0.38 (0.25, 0.58), 0.61 (0.46, 0.80), and 0.74 (0.55, 0.99), and for TTLSP, 0.79 (0.45, 1.36), 0.65 (0.42, 1.00), 1.01 (0.66, 1.55). A significant negative association between nutrition knowledge and ITP was also observed: ORs with 95% CIs for ADP from “Low nutrition knowledge” to “Moderate nutrition knowledge” were 8.41 (5.36, 13.21) and 1.92 (1.27, 2.89); for ITP were 1.93 (1.41, 2.65) and 1.19 (0.96, 1.47); and for TTLSP were 1.62 (1.01, 2.59) and 1.17 (0.84, 1.64). Our analysis did not find significant association between smoking, sex, or physical activity and EF pattern.

**Figure 2:**
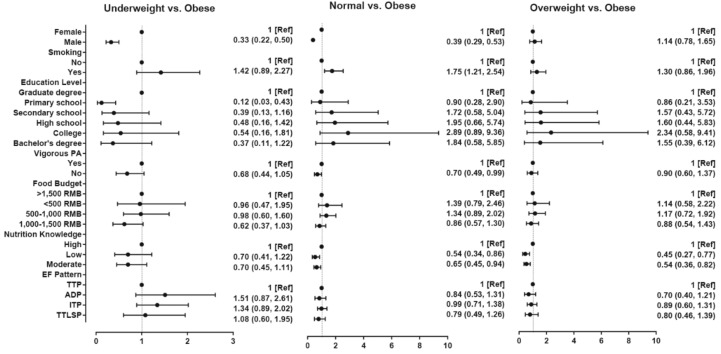
Associations between nutrition knowledge, sociodemographic, and lifestyle factors, EF, and body mass index (BMI) group.

Model 2 utilized BMI group as the dependent variable (Figure 2 and Table S2); the results showed that people who smoked were more likely to have a normal BMI compared with those who did not (OR with 95% CI: 1.75 (1.21, 2.54)). Less vigorous physical activity was significantly associated with lower odds of being normal weight (OR with 95% CI: 0.70 (0.49, 0.99)). Men were less likely than women to be underweight or normal weight (OR with 95% CI for underweight: 0.33 (0.22, 0.50); for normal weight: 0.39 (0.29, 0.53)). People with lower nutrition knowledge were less likely to be normal weight or overweight. ORs with 95% CIs for normal and underweight among people with low nutrition knowledge were respectively 0.54 (0.34, 0.86) and 0.45 (0.27, 0.77), and among people with moderate nutrition knowledge, 0.65 (0.45, 0.94) and 0.54 (0.36, 0.82). No significant association between food budget and BMI group was observed in our results.

## 4. Discussion

In this study, we found that lower education level, a larger food budget, and lower nutrition knowledge were associated with higher likelihood of ITP. In addition, women and participants who reported smoking, more vigorous physical activity, higher education level, and higher nutrition knowledge were less likely to be obese. It is noteworthy that the significant association between EF patterns and BMI group disappeared in the regression model after adjustment for age, sex, education, smoking, food budget, nutrition knowledge, and physical activity, suggesting that it was these factors that impacted obesity and that EF pattern might just be a manifestation of these characteristics.

Previous studies have reported significant associations between EF and many sociodemographic and lifestyle factors, such as age, sex, education, smoking status, physical activity, and food budget [10,26,33,34,35,36], though few investigations have relied on models to predict the strength and direction of these associations. Consistent with the present findings, sociodemographic and lifestyle factors (e.g., physical activity) have previously been shown to contribute to the development of obesity [1]. For example, the prevalence of obesity shows significant variation by age and sex groups in many national surveys [28,29,37], and higher education level is often associated with a lower risk of obesity [24,38]. Inverse associations of nutrition knowledge and smoking with BMI, waist circumference, and waist circumference/height ratio have also been previously reported [27,38]. Higher nutrition knowledge might decrease the risk of obesity [25,26], consistent with our present results.

Contrary to some previously reported research conducted in Western contexts, however, the relationship between EF pattern and obesity in the present study was no longer statistically significant after adjustment for confounding factors. However, this finding is consistent with the results of a cohort study conducted by Kant et al. [16], whereas Duval et al. similarly found that the association between EF and BMI to be non-significant after adjustment for energy expenditure and physical fitness [17]. A randomized controlled study performed by Huseinovic et al. suggested that EF during intervention had a crude association with weight gain, but this association turned to be non-significant after adjustment for physical activity level, EF at baseline, and dietary treatment [15]. Nevertheless, other researchers have also found an inverse association [9,10,11,13,14,33] or positive relationship [18,19] between EF and BMI; however, they either did not take confounding factors into consideration or included fewer confounding factors than the present study.

The difference in EF definition might also contribute to inconsistent results. There are four definitions of EF applied in current studies: (1) contribution to total energy intake [20], (2) time of day [21], (3) self-reported eating occasions [22], and (4) neutral definition [23]. Among studies which focus on the association between EF and obesity, five studies calculated EF with time of day [10,14,16,17,19], three with contribution to energy intake [15,18,33], two with self-reported eating occasions [9,11], and one with neutral definition [13]. The methodological difference in the definition is likely to impact reported EF, but our results are consistent with other studies that using different EF definitions [15,16,17].

The fundamental modifiable cause of obesity is energy imbalance. Some studies have previously reported an inverse relationship between EF and energy intake [39,40], but overall, the research was equivocal [41]. In a randomized cross-over study, for instance, reduced EF did not have a significant impact on energy expenditure [10], and studies examining 24-h energy expenditure failed to detect differences between different EF groups [42]. Thus, EF does not appear to influence obesity in humans based on existing evidence; it might just be a symptom or by-product of associations among nutrition knowledge, sociodemographic, and lifestyle factors and obesity.

This study has several limitations, however. First, the cross-sectional design does not allow for the inference of causal relationships. Second, most of the data collected for this study were self-reported and, thus, vulnerable to recall bias, and the measures were potentially influenced by social norms. Third, ours is a convenience sample, which may limit generalizability. In addition, budgetary constraints limited data collection to one 24-hour dietary recall for estimating EF. Furthermore, a large time interval criterion may not be able to capture smaller eating occasions such as beverage-only occasions. A prospective cohort study in a Chinese population using objective, validated measures would improve the quality of data and impart a deeper understanding of the associations among EF, nutrition knowledge, sociodemographic, and lifestyle factors and obesity.

## 5. Conclusions

Our findings suggested that EF is associated with sociodemographic and lifestyle factors but not with obesity. The difference in BMI group among EF patterns might be just an artifact of the relationships between sociodemographic and lifestyle factors and obesity.

## Figures and Tables

**Table 1:** Characteristics of the study population (*N* = 2290).

Characteristics	Male (*N* = 1162)		Female (*N* = 1128)		Total (*N* = 2290)	
	*N*	%	*N*	%	*N*	%
Age group						
29–34	27	2%	40	4%	67	3%
34–39	158	14%	284	25%	442	19%
39–44	530	46%	545	48%	1075	47%
44–49	356	31%	213	19%	569	25%
49–54	70	6%	32	3%	102	4%
54–59	13	1%	6	1%	19	1%
59–64	5	0%	3	0%	8	0%
64–69	2	0%	4	0%	6	0%
69–74	1	0%	1	0%	2	0%
Highest level of education						
Primary school	49	4%	68	6%	117	5%
Secondary school	420	36%	409	36%	829	36%
High school	442	38%	430	38%	872	38%
College	117	10%	108	10%	225	10%
Bachelor’s degree	111	10%	94	8%	205	9%
Graduate degree	23	2%	19	2%	42	2%
Smoking						
Yes	548	47%	33	3%	581	25%
No	614	53%	1095	97%	1709	75%
Food budget monthly						
Less than 500 RMB	141	12%	93	8%	234	10%
500–1000 RMB	474	41%	504	45%	978	43%
1000–1500 RMB	350	30%	338	30%	688	30%
More than 1500 RMB	197	17%	193	17%	390	17%
Vigorous PA (4+ days)						
No	878	76%	916	81%	1794	78%
Yes	284	24%	212	19%	496	22%
Nutrition knowledge						
Low	220	19%	168	15%	388	17%
Moderate	659	57%	624	55%	1283	56%
High	283	24%	336	30%	619	27%
BMI Group						
Normal	613	53%	724	64%	1337	58%
Obese	147	13%	87	8%	234	10%
Overweight	278	24%	134	12%	412	18%
Underweight	124	11%	183	16%	307	13%
EF pattern						
ADP	12.31%	143	10.82%	122	265	12%
TTLSP	10.93%	127	9.66%	109	236	10%
ITP	28.66%	333	31.38%	354	687	30%
TTP	48.11%	559	48.13%	543	1102	48%

**Table 2:** Differences in body mass index (BMI) distribution among eating frequency (EF) patterns.

	Underweight		Normal		Overweight		Obese		All
	*N*	%	*N*	%	*N*	%	*N*	%	*N*
EF Pattern									
ADP	53	20.00%	139	52.45%	39	14.72%	34	12.83%	265
TTLSP	33	13.98%	132	55.93%	44	18.64%	27	11.44%	236
ITP	104	15.14%	400	58.22%	115	16.74%	68	9.90%	687
TTP	117	10.62%	666	60.44%	214	19.42%	105	9.53%	1102
All	307	13.41%	1337	58.38%	412	17.99%	234	10.22%	2290

χ^2^ = 25.40, *p* = 0.003.

**Table 3:** Differences in nutrition knowledge distribution among EF patterns.

	Low		Moderate		High		All
	*N*	%	*N*	%	*N*	%	*N*
EF Pattern							
ADP	110	41.51%	121	45.66%	34	12.83%	265
ITP	122	17.76%	389	56.62%	176	25.62%	687
TTLSP	36	15.25%	137	58.05%	63	26.69%	236
TTP	120	10.89%	636	57.71%	346	31.40%	1102
All	388	16.94%	1283	56.03%	619	27.03%	2290

χ^2^ = 152.81, *p* < 0.001.

**Table 4:** Differences in food budget distribution among EF patterns.

	Less than 500 RMB		500–1000 RMB		1000–1500 RMB		More than 1500 RMB		All
	N	%	N	%	N	%	N	%	N
EF Pattern									
ADP	27	10.19%	75	28.30%	96	36.23%	67	25.28%	265
ITP	48	6.99%	295	42.94%	207	30.13%	137	19.94%	236
TTLSP	27	11.44%	89	37.71%	81	34.32%	39	16.53%	687
TTP	132	11.98%	519	47.10%	304	27.59%	147	13.34%	1102
All	27	10.19%	75	28.30%	96	36.23%	67	25.28%	2290

χ^2^ = 59.83, *p* < 0.001.

## Supplementary Materials

The following are available online at http://www.mdpi.com/1660-4601/15/11/2561/s1, Table S1. Associations between nutrition knowledge, sociodemographic and lifestyle factors and EF, Table S2. Associations between nutrition knowledge, sociodemographic and lifestyle factors, EF, and BMI group.

## Abbreviations

EF: eating frequency; BMI: body mass index; OR: odds ratio; ADP: all-day pattern; TTPLSP: traditional time plus late snack pattern; ITP: irregular time pattern; TTP: traditional time pattern.
