# Supplementary material for: Eating Frequency Is Not Associated with Obesity in Chinese Adults
Source: Int J Environ Res Public Health. 2018 Nov 15;15(11):2561. doi: 10.3390/ijerph15112561 (PMC6266818; doi:10.3390/ijerph15112561)
Supplement: Supplementary file 1 [file ijerph-15-02561-s001.pdf]

International Journal of
Environmental Research and Public Health

MDPI

Table S1. Associations between nutrition knowledge, sociodemographic and lifestyle factors and EF.

	ADP vs. TTP		ITP vs. TTP		TTLSP vs. TTP	
	**OR (95% CI)**	**P**	**OR (95% CI)**	**P**	**OR (95% CI)**	**P**
Smoking						
No	1 [Ref]		1 [Ref]		1 [Ref]	
Yes	1.14 (0.78, 1.65)	0.47	1.07 (0.83, 1.38)	0.61	1.36 (0.94, 1.98)	0.10
Highest level of education						
Graduate degree	1 [Ref]		1 [Ref]		1 [Ref]	
Primary school	0.20 (0.07, 0.56)	**0.003** *	0.27 (0.10, 0.73)	**0.009** *	0.13 (0.03, 0.64)	**0.011** *
Secondary school	0.20 (0.08, 0.52)	**<0.001** **	0.47 (0.19, 1.13)	0.09	0.51 (0.15, 1.74)	0.28
High school	0.17 (0.07, 0.43)	**<0.001** **	0.39 (0.16, 0.95)	**0.039** *	0.49 (0.14, 1.67)	0.25
College	0.11 (0.04, 0.32)	**<0.001** **	0.28 (0.11, 0.69)	**0.006** *	0.44 (0.12, 1.59)	0.22
Bachelor's degree	0.10 (0.03, 0.29)	**<0.001** **	0.28 (0.11, 0.70)	**0.007** *	0.26 (0.07, 0.99)	**0.047** *
Vigorous PA (4+ days)						
Yes	1 [Ref]		1 [Ref]		1 [Ref]	
No	1.23 (0.87, 1.76)	0.23	1.11 (0.87, 1.40)	0.39	1.15 (0.81, 1.64)	0.43
Food budget monthly (RMB)						
>1500	1 [Ref]		1 [Ref]		1 [Ref]	
<500	0.46 (0.27, 0.80)	**0.0053** *	0.38 (0.25, 0.58)	**<0.001** **	0.79 (0.45, 1.36)	0.40
500–1000	0.36 (0.24, 0.54)	**<0.001** **	0.61 (0.46, 0.80)	**<0.001** **	0.65 (0.42, 1.00)	**0.047** *
1000–1500	0.79 (0.54, 1.18)	0.26	0.74 (0.55, 0.99)	**0.047** *	1.01 (0.66, 1.55)	0.98
Sex						
Female	1 [Ref]		1 [Ref]		1 [Ref]	
Male	0.98 (0.70, 1.37)	0.90	0.89 (0.70, 1.12)	0.29	0.95 (0.68, 1.33)	0.79
Nutrition knowledge						
High	1 [Ref]		1 [Ref]		1 [Ref]	
Low	8.41 (5.36, 13.21)	**<0.001** **	1.93 (1.41, 2.65)	**<0.001** **	1.62 (1.01, 2.59)	**0.046** *
Moderate	1.92 (1.27, 2.89)	**0.002** *	1.19 (0.96, 1.47)	0.13	1.17 (0.84, 1.64)	0.34

*: $p < 0.05$; **: $p < 0.01$.

Table S2. Associations between nutrition knowledge, sociodemographic and lifestyle factors, EF, and BMI group.

	**Underweight vs. Obese**		**Normal vs. Obese**		**Overweight vs. Obese**	
	**OR (95% CI)**	**P**	**OR (95% CI)**	**P**	**OR (95% CI)**	**P**
Smoking						
No	1 [Ref]		1 [Ref]		1 [Ref]	
Yes	1.42 (0.89, 2.27)	0.15	1.75 (1.21, 2.54)	**0.003** *	1.30 (0.86, 1.96)	0.21
Highest level of education						
Graduate degree	1 [Ref]		1 [Ref]		1 [Ref]	
Primary school	0.12 (0.03, 0.43)	**0.001** *	0.90 (0.28, 2.90)	0.85	0.86 (0.21, 3.53)	0.84
Secondary school	0.39 (0.13, 1.16)	0.09	1.72 (0.58, 5.04)	0.33	1.57 (0.43, 5.72)	0.49
High school	0.48 (0.16, 1.42)	0.18	1.95 (0.66, 5.74)	0.22	1.60 (0.44, 5.83)	0.48
College	0.54 (0.16, 1.81)	0.32	2.89 (0.89, 9.36)	0.08	2.34 (0.58, 9.41)	0.23
Bachelor's degree	0.37 (0.11, 1.22)	0.10	1.84 (0.58, 5.85)	0.30	1.55 (0.39, 6.12)	0.53
Vigorous PA (4+ days)						
Yes	1 [Ref]		1 [Ref]		1 [Ref]	
No	0.68 (0.44, 1.05)	0.08	0.70 (0.49, 0.99)	**0.0498** *	0.90 (0.60, 1.37)	0.63
Food budget monthly (RMB)						
>1500	1 [Ref]		1 [Ref]		1 [Ref]	
<500	0.96 (0.47, 1.95)	0.92	1.39 (0.79, 2.46)	0.26	1.14 (0.58, 2.22)	0.69
500–1000	0.98 (0.60, 1.60)	0.95	1.34 (0.89, 2.02)	0.18	1.17 (0.72, 1.92)	0.52
1000–1500	0.62 (0.37, 1.03)	0.06	0.86 (0.57, 1.30)	0.48	0.88 (0.54, 1.43)	0.60
Sex						
Female	1 [Ref]		1 [Ref]		1 [Ref]	
Male	0.33 (0.22, 0.50)	**<0.001** **	0.39 (0.29, 0.53)	**<0.001** **	1.14 (0.78, 1.65)	0.50
Nutrition knowledge						
High	1 [Ref]		1 [Ref]		1 [Ref]	
Low	0.70 (0.41, 1.22)	0.21	0.54 (0.34, 0.86)	**0.009** *	0.45 (0.27, 0.77)	**0.004** *
Moderate	0.70 (0.45, 1.11)	0.12	0.65 (0.45, 0.94)	**0.023** *	0.54 (0.36, 0.82)	**0.004** *
EF pattern						
TTP	1 [Ref]		1 [Ref]		1 [Ref]	
ADP	1.51 (0.87, 2.61)	0.14	0.84 (0.53, 1.31)	0.44	0.70 (0.40, 1.21)	0.20
ITP	1.34 (0.89, 2.02)	0.17	0.99 (0.71, 1.38)	0.94	0.89 (0.60, 1.31)	0.55
TTLSP	1.08 (0.60, 1.95)	0.79	0.79 (0.49, 1.26)	0.33	0.80 (0.46, 1.39)	0.43

*: $p < 0.05$; **: $p < 0.01$.
